# Challenges in Grisel's Syndrome Management in a Two-Month-Old Infant

**DOI:** 10.7759/cureus.35128

**Published:** 2023-02-17

**Authors:** Maeen B Aldamouni, Mohammed H Albitar, Ziad H Alhosainy, Hanan N Aljohani, Essam Alshail

**Affiliations:** 1 Department of Neurosurgery, King Faisal Specialist Hospital and Research Centre, Riyadh, SAU; 2 College of Medicine, Alfaisal University, Riyadh, SAU

**Keywords:** pediatric neurosurgery, torticollis, retropharyngeal abscess, nontraumatic atlantoaxial subluxation, grisel syndrome

## Abstract

Grisel’s syndrome (GS) is a rare neurosurgical condition involving nontraumatic rotatory subluxation of the atlantoaxial joint. This case report presents a two-month-old infant girl, the youngest reported case of this syndrome based on our literature review to the date of this publication. The infant was initially referred to our hospital as a case of the arachnoid cyst but was subsequently neuroradiologically diagnosed with GS, which was believed to be secondary to a retropharyngeal abscess. After developing weakness and developmental delay as well as failing conservative management for two years, the infant underwent C1 laminectomy and occipitocervical sublaminar wire fusion with favorable outcomes. GS should be considered a differential even if the patient does not present with typical signs such as torticollis and neck pain. If not identified early and treated effectively, it can result in severe neurological damage. The management plan largely depends on the Fielding-Hawkins grade of subluxation and the timing of diagnosis.

## Introduction

Grisel’s syndrome (GS) is a rare condition characterized by nontraumatic rotatory atlantoaxial subluxation (AAS) of the atlantoaxial joint (C1-C2) [1]. The first case was described in 1830 by Sir Charles Bell in a patient who had subluxation along with spinal cord compression as a result of a syphilitic pharyngeal ulcer [2]. A century later, the condition was named after Pierre Grisel after two patients were reported with AAS following tonsillectomy and nasopharyngitis [3,4]. The syndrome may occur after upper respiratory tract infections such as pharyngitis, otitis, and mastoiditis; head and neck infections such as lymphadenitis and retropharyngeal abscess; or otolaryngology procedures such as tonsillectomy, adenoidectomy, mastoidectomy, cochlear implantation, uvulectomy, and tympanoplasty [5]. Other rare causes that have been reported include mumps, tuberculosis, and Kawasaki disease [1].

GS predominantly affects young patients, usually those under 12, and is infrequently reported in adults [6,7]. The presentation is variable, but typically patients would present with torticollis, restricted head movement, pain, and sometimes difficulty swallowing [8]. History, physical examination, and neuroradiological workup using X-rays, computed tomography (CT), or magnetic resonance imaging (MRI) of the cervical spine are used to aid in the diagnosis [9,10].

No universal treatment algorithm exists. However, conservative management such as antibiotics, muscle relaxants, analgesics, and cervical collars are usually regarded as the first-line treatment option, while surgery is indicated for advanced cases: failed conservative treatment, recurrence of subluxation, or irreducible subluxations [6,11]. We present the youngest case of GS, who developed AAS following a retropharyngeal abscess and had failed different conservative management; thus, the patient had to undergo surgical fixation, which was challenging due to the young age and weak bones.

## Case presentation

Patient history and first admission

We report a case of a two-month-old girl who was referred to King Faisal Specialist Hospital and Research Centre (KFSH&RC), Riyadh, Saudi Arabia, from a peripheral hospital in Jizan due to a quadrigeminal cistern arachnoid cyst. Scans done showed that it was causing compression to the pineal region and subsequent hydrocephalus and dilatation of the third and lateral ventricles. The patient was delivered via C-section and was diagnosed with the cyst at birth. She had a history of fever and meningitis that was treated with IV ceftriaxone when she was 16 days old. At the time of presentation to the emergency department in KFSH&RC, she appeared pale and cyanosed, had high blood pressure and a normal but irregular breathing rate, and did not have a fever. The cyanosis episodes with low oxygen saturation would occur whenever the patient cries or is fed.

**Figure 1:**
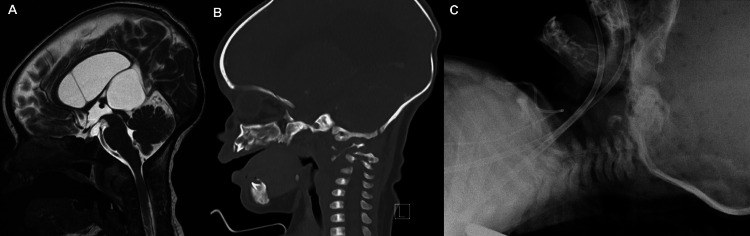
At first presentation. (A) T2-weighted brain image shows abscess formation involving the C1-C2 level with prevertebral enhanced soft tissue inflammatory changes. Significant bone destruction on the right side of C1-C3 and narrowing of the craniocervical junction are also evident. (B) Head CT shows the phlegmonous reaction in the retropharyngeal space with definite residual abscess collection as well as widening of predental space with atlantoaxial subluxation. (C) Cervical X-ray demonstrates widened atlanto-odontoid interval, basilar invagination of the upper cervical spine, and pre-atlantoaxial retropharyngeal soft tissue thickening.

Initial neuroradiology scans were done and showed incidental findings of an upper cervical spondylodiscitis with a convex-shaped fluid collection of anterior and posterior craniocervical ligaments that caused moderate narrowing of the craniocervical junction and compression of the upper cervical spine on CT (Figure 1). Despite the patient being afebrile, there were signs suggestive of a retropharyngeal abscess. Initial MRI scans done on day two postadmission confirmed a massive retropharyngeal abscess along with signs of atlantoaxial subluxation; the abscess showed improvement in size two months postadmission. Due to this improvement and her young age, the ENT team decided that the patient was not a candidate for surgical drainage of the abscess. The patient also had bacteremia indicated by positive blood culture for coagulase-negative staphylococci contaminants, specifically *Staphylococcus epidermidis* and *Staphylococcus hominis*. Thus, intravenous (IV) vancomycin and meropenem were administered to treat the retropharyngeal abscess and the bacteremia.

The patient had dysphagia and reflux, so she was on nasogastric tube feeding and periodically visited a speech-language pathologist. She also underwent extensive investigation by pediatric allergy immunology to rule out any immunodeficiency. It was noticed that the patient had decreased shoulder and hip movement, so an ultrasound (US) and X-ray for both were done, which revealed developmental dysplasia of the hip (DDH) and asymmetrical humerus head ossification, after which she was referred to pediatric orthopedics. In less than a year, her DDH was resolved.

In this patient, GS was first diagnosed two weeks postadmission when repeat scans showed definitive signs of the syndrome. Due to dysphagia worsening and craniocervical narrowing during her admission, she was put on a cervical brace that was modified by occupational therapy for her unstable craniocervical junction. The patient was kept in this brace throughout her admission and was discharged later.

Follow-up at one year of age

A follow-up at 11 months with repeat scans showed a stable cyst along with subluxation. Two months later, the patient was admitted again, and a flexion-extension X-ray was done, which revealed a deformed C2 vertebra with more sclerosis, widening of the atlanto-odontoid interval measuring 6 mm, and similar findings as of her presentation. The pediatric neurosurgery team discussed the best suitable surgical approach and contacted orthotics to design a custom cervical-thoracic-lumbar-sacral orthosis (CTLSO), as ready-made instruments are not available for the patient’s age. Three weeks after her discharge, she had an appointment with the pediatric physiotherapist, where she was found to have developmental delay and muscle weakness and was advised to do therapeutic and bed mobility exercises.

Follow-up at two years of age

**Figure 2:**
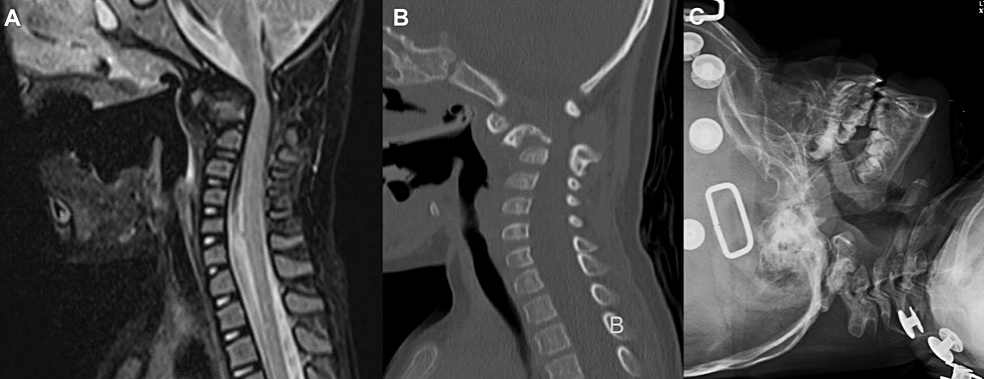
Preoperative. (A) T2 MRI brain showing atlantoaxial subluxation worsening. (B) Neck CT demonstrating stable destructive changes involving atlantoaxial level and causing narrowing and kinking of the spinal cord. (C) Neck X-ray showing a deformed C2 vertebra with a further widening of the atlanto-odontoid interval and narrowing of the foramen magnum. MRI, magnetic resonance imaging; CT, computed tomography

She got admitted again at 23 months due to subluxation worsening along with hemiplegia. Routine labs were normal, and neuroradiology scans were done (Figure 2). On redemonstration of bone formation, an abnormal configuration of C1-C2 anterior dislocation was felt. There was also a presence of a C2-C3 posterior element. The pediatric neurosurgical team decided for the patient to undergo surgery, given that there was no improvement with the CTLSO and as the patient was two years old.

The patient underwent an occipitocervical fixation with C1 laminectomy to decompress the spinal cord and an occipitocervical C2-C3 sublaminar wire fusion with bone graft between the C2 and suboccipital bone. During the surgery, when applying the tie to the opening drilled through the suboccipital bone, a fracture occurred through the opening. For that, a four-hole plate was applied with screws to overcome the fracture, and then a wire tie was introduced through the burr hole. While the wire was inserted near the spinal cord, the patient went bradycardic and suddenly developed asystole, so cardiopulmonary resuscitation (CPR) was done for 30 seconds. There was no desaturation, and epinephrine was not required during the arrest. EEG recording during this event showed no change or abnormality, indicating no ischemia. The event was likely secondary to a vasovagal attack due to the wire touching and stimulating the spinal cord. Neurophysiology recording was at the baseline throughout the procedure, with normal motor evoked potential and an absence of somatosensory evoked potential since the beginning of the procedure.

**Figure 3:**
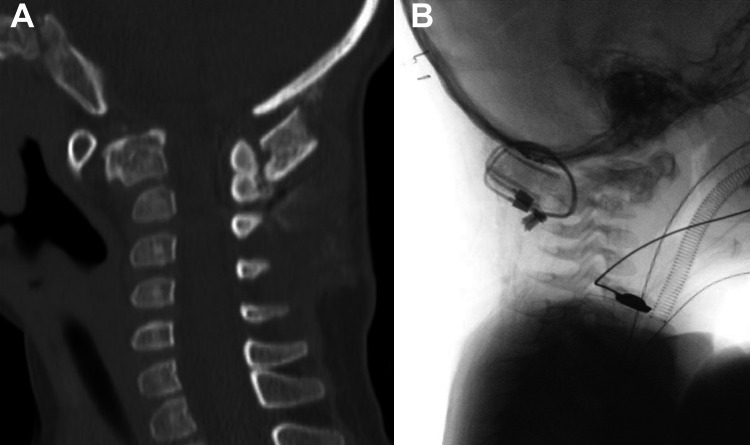
Postoperative. (A) Neck CT showing interval wire fusion of the occiput and upper cervical spine with the significantly improved caliber of the spinal canal and reduced spinal cord compression. (B) Craniocervical junction fluoroscopy demonstrating interval posterior stabilization of the skull base and C1-C2 posterior elements by cerclage wires to correct for the atlantoaxial instability.

Neuroradiology scans were done after the surgery and showed stable postop findings (Figure 3). The patient had her collar adjusted by orthotics and was discharged within a week after the surgery. She was recommended to undergo intensive rehabilitation after recovery once there were no movement restrictions to enhance the developmental milestone as per her age.

Follow-up visits

**Figure 4:**
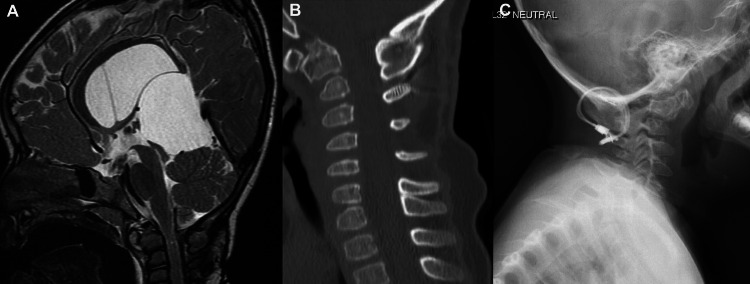
Last follow-up. (A) T2-weighted brain MRI shows significant improvement of the craniocervical junction narrowing along with focal atrophy and myelomalacia at the C1-C2 level. (B) The craniocervical junction CT demonstrates bony fusion with posterior assimilation at the occiput through C3 with intact cerclage wires. (C) Neck X-ray reveals a persistent increase in C1 and C2 vertebral body sclerosis with residual mild anterior translation. The atlantodental interval is significantly narrowed as well. MRI, magnetic resonance imaging

She was seen twice after surgery - at two weeks and nine weeks postsurgery. During these appointments, the wound was healing well, and she could move all limbs and sit. The patient was able to ambulate with assistance at 14 months after the surgery when she was aged about three years but could not stand without assistance. She had bilateral lower limb weakness, mild lower limb hyporeflexia, and limited head movement to the right side, for which she was recommended to undergo extensive physical therapy. Radiology scans of the spine at that time showed significantly improved atlantoaxial interval and compression of the spinal canal (Figure 4). At her latest follow-ups at four and five years of age, she had stable findings, was able to stand and walk alone, had baseline ataxia, and had significantly improved range of motion after the physical therapy, but with difficulty turning her head to both sides that were more pronounced on the right side.

## Discussion

GS is a rare clinical entity predominantly seen in childhood. The youngest age reported in the literature is six months at the time of diagnosis, making our case the youngest [12]. Her infancy presented several challenges. The decision not to drain the abscess came from the ENT team due to her young age and her good response to antibiotics. Also, her age made her present atypically with no typical signs and symptoms of the syndrome, such as torticollis, difficulty in tilting the head, cervical pain, and neurologic impairment [13]. Because the diagnosis of GS in children is often overlooked, it is crucial to suspect it in children after head and neck infections, even if they are not presenting with classical signs due to their young age, as early diagnosis and treatment are critical to prevent any neurological complications [10].

The mechanism of GS pathogenesis has not been completely elucidated despite multiple proposed theories over the years [12]. However, the two-hit hypothesis proposed by Battiata et al. seems the most plausible [14]. The first hit would be the preexisting laxity of the cervical ligaments and atlantoaxial joint instability, which are usually seen in the pediatric age group. The second hit would be the transfer of the inflammatory exudate from the peripharyngeal infection to the cervical muscles and ligaments through the pharyngovertebral venous plexus, leading to spasm and laxity with resultant subluxation [14]. In our case, we believe that a combination of multiple factors led to the syndrome. First, there is likely a preexisting ligamentous laxity due to the infant’s very young age. Second, the bacterial spread to the joint is most likely from the retropharyngeal abscess, which was likely caused by the previous bacteremia the patient had that was treated. The retropharyngeal abscess was either due to the previous upper respiratory tract infection the patient had or the previous middle finger osteomyelitis that the mother reported.

As our patient was originally admitted as a case of an arachnoid cyst and the retropharyngeal abscess was picked up incidentally while evaluating the cyst, the diagnosis was delayed and led to the spread of infection from the retropharyngeal abscess to the dens process and prevented it from growing normally. By the time the infection resolved, repeat scans had shown a damaged dens process, which affected the growth of her cervical spine, as evident by the difficulty in turning the head several years after successful surgery.

The neuroradiological examination of GS includes X-ray, CT scan, and MRI. According to a recent review by Wenger et al., contrast-enhanced MRI should be considered the primary diagnostic imaging modality in patients suspected to have GS based on history and clinical presentation [10]. MRI would usually reveal the normal soft tissue, lymph nodes, ligamentous laxity of the transverse and alar ligaments, and any dislocation or cord compression [7]. The treatment paradigm for GS is still controversial, and there are no clear management guidelines. Two factors might play a role in the decision of the treatment plan, the Fielding-Hawkins grade of subluxation and the timing of the diagnosis and treatment [5,7].

Type I and type II are the most common and are generally treated conservatively with antibiotics, muscle relaxants, anti-inflammatory drugs, and bed rest. For type 1, a soft cervical collar can be used for four weeks, while for type II, a hard collar can be used for up to eight weeks [5,7]. Another treatment used for both type I and type II is a reduction with halter traction followed by rigid immobilization. Although it has high success rates, it is associated with multiple complications [15]. In case of neurological impairment or treatment failure, more aggressive management can be considered. Fielding’s type III and type IV subluxations are more likely to be associated with neurological deficits. Thus, more invasive approaches such as halo immobilization, arthrodesis, and C1-C2 cervical fusion might be deployed [9,16]. Nonetheless, all these techniques might not apply to each case, and thus, patients should be individually managed.

This case had an atlanto-odontoid interval measured as 5 mm, corresponding to Fielding type I subluxation [4]. With the failure of conservative management offered to her, likely due to her young age and fragile bones, surgery with C1 laminectomy and occipitocervical C2-C3 sublaminar wire fusion with bone graft was ultimately needed, but it was delayed for two years until the bone had ossified.

Pathogens implicated in GS are rarely identified, but those that have been reported include *Streptococcus pyogenes, Bacteroides ureolyticus, Mycobacterium tuberculosis, Pseudomonas aeruginosa, Staphylococcus aureus*, methicillin-resistant *S. epidermidis*, and Epstein-Barr virus [17]. We report the first case in which *S. hominis* was isolated.

## Conclusions

We reported the youngest case of GS, highlighting the challenges faced in GS management due to the patient's young age and the complications that arose due to the delayed diagnosis. Findings from this report support the evidence that conservative management has limited success when the diagnosis is delayed, the patient has an advanced Fielding-type subluxation, or the patient is young. Therefore, it is important to consider GS as a differential diagnosis if the patient presents with fever and other related signs after an otolaryngology procedure, head and neck infection, or upper respiratory tract infection. With early diagnosis and treatment, permanent complications can be avoided and less invasive treatment would be needed.
